# Modulation of gut microbiota by probiotics to improve the efficacy of immunotherapy in hepatocellular carcinoma

**DOI:** 10.3389/fimmu.2024.1504948

**Published:** 2024-11-22

**Authors:** Ping Chen, Chengchen Yang, Ke Ren, Mingzhi Xu, Chenwei Pan, Xuewei Ye, Lanjuan Li

**Affiliations:** ^1^ Key Laboratory of Artificial Organs and Computational Medicine of Zhejiang Province, Shulan (Hangzhou) Hospital, Shulan International Medical College, Zhejiang Shuren University, Hangzhou, China; ^2^ State Key Laboratory for Diagnosis and Treatment of Infectious Diseases, First Affiliated Hospital, School of Medicine, Zhejiang University, Hangzhou, China; ^3^ Shulan International Medical College, Zhejiang Shuren University, Hangzhou, China; ^4^ Department of General Medicine, Zhejiang Cancer Hospital, Institute of Basic Medicine and Cancer (IBMC), Chinese Academy of Sciences, Hangzhou, Zhejiang, China; ^5^ Department of Infectious Diseases, The Second Affiliated Hospital of Wenzhou Medical University, Wenzhou, China; ^6^ Key Laboratory of Pollution Exposure and Health Intervention of Zhejiang Province, Shulan International Medical College, Zhejiang Shuren University, Hangzhou, China; ^7^ National Clinical Research Center for Infectious Diseases, First Affiliated Hospital, School of Medicine, Zhejiang University, Hangzhou, China

**Keywords:** probiotics, gut microbiota, hepatocellular carcinoma, immunotherapy, therapeutic strategies

## Abstract

Hepatocellular carcinoma, a common malignancy of the digestive system, typically progresses through a sequence of hepatitis, liver fibrosis, cirrhosis and ultimately, tumor. The interaction between gut microbiota, the portal venous system and the biliary tract, referred to as the gut-liver axis, is crucial in understanding the mechanisms that contribute to the progression of hepatocellular carcinoma. Mechanisms implicated include gut dysbiosis, alterations in microbial metabolites and increased intestinal barrier permeability. Imbalances in gut microbiota, or dysbiosis, contributes to hepatocellular carcinoma by producing carcinogenic substances, disrupting the balance of the immune system, altering metabolic processes, and increasing intestinal barrier permeability. Concurrently, accumulating evidence suggests that gut microbiota has the ability to modulate antitumor immune responses and affect the efficacy of cancer immunotherapies. As a new and effective strategy, immunotherapy offers significant potential for managing advanced stages of hepatocellular carcinoma, with immune checkpoint inhibitors achieving significant advancements in improving patients’ survival. Probiotics play a vital role in promoting health and preventing diseases by modulating metabolic processes, inflammation and immune responses. Research indicates that they are instrumental in boosting antitumor immune responses through the modulation of gut microbiota. This review is to explore the relationship between gut microbiota and the emergence of hepatocellular carcinoma, assess the contributions of probiotics to immunotherapy and outline the latest research findings, providing a safer and more cost-effective potential strategy for the prevention and management of hepatocellular carcinoma.

## 1 Introduction

Hepatocellular carcinoma (HCC), recognized as the leading form of liver cancer, is a prevalent malignancy of the digestive system and represents a significant global health challenge (1). HCC is among the top six cancers diagnosed around the world and stands as a major contributor to cancer mortality, underscoring the urgent need for improved preventive and therapeutic strategies (2). In recent years, immunotherapy for HCC has made significant strides, with an increasing number of immunotherapeutic agents emerging as viable first- and second-line treatment options. These therapies have demonstrated efficacy in controlling tumor progression and extending patient survival (3, 4).

Immune checkpoint inhibitors (ICIs) work by reversing tumor-induced immune suppression in HCC, thereby restoring and enhancing the T cells’ ability to target and eliminate cancer cells. Currently, the most widely used drugs in liver cancer immunotherapy are programmed cell death protein 1(PD-1) and programmed cell death-Ligand 1(PD-L1) inhibitors. They block the interaction between PD-1 and PD-L1, thereby reactivating tumor-specific T cells, enhancing their cytotoxic function, and promoting the destruction of tumor cells. Once the anti-tumor immune cycle is established, a sustained immune response can be achieved. Substantial evidence has demonstrated the association between gut microbiota and cancer (5). Gut microbiota is pivotal in regulating responses to cancer immunotherapy (6–11), while the microbial communities within the tumor microenvironment (TME) have been shown to enhance therapeutic efficacy (12).

**Figure 1:**
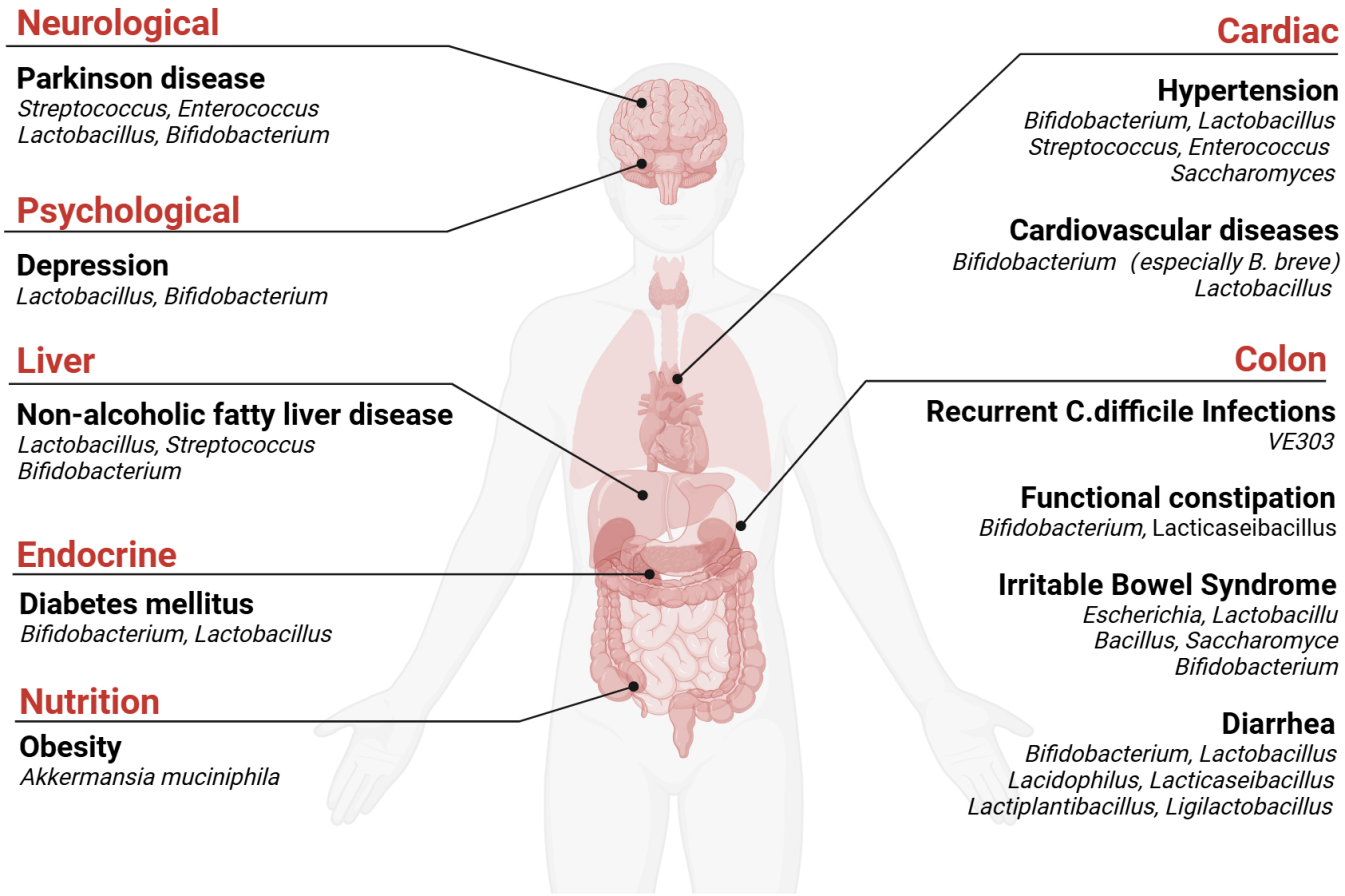
Clinical application of probiotics Created in BioRender. c, c. (2024) https://BioRender.com/z26s686.

Probiotics are preparations formulated using the principles of microecology, incorporating beneficial probiotics or growth-promoting substances that are harmless to the host, produced through specialized processes. These preparations can function as biochemical barriers, improving liver and intestinal function and enhancing immune responses. Probiotics have demonstrated wide-ranging clinical applications (**Figure 1**), such as treating recurrent Clostridium difficile infection (CDI) (13), alleviating functional constipation (14), managing non-alcoholic fatty liver disease (NAFLD) (15) and so on (16–19). Additionally, probiotics can be utilized to regulate blood pressure (20), modulate blood lipid levels (21), alleviate gastrointestinal symptoms in patients with Parkinson disease and indirectly impact motor symptoms, and improve Beck Depression Inventory (BDI) scores in individuals with major depressive disorder (22–24). Emerging evidence suggests that probiotics may enhance the efficacy of HCC immunotherapy through gut microbiota modulation, representing a promising novel therapeutic approach for HCC management.

## 2 The relationship between gut microbiota and HCC

### 2.1 Gut-liver axis and HCC

The gut-liver axis, a bidirectional communication system formed by gut microbiota, the portal venous system and the biliary tract, underlies the physiological interactions between gut microbiota and the liver (24). Originally, it described the immune response in cirrhotic patients to gut-derived microbes and food antigens in the bloodstream. Over the past decade, this concept has expanded to include a variety of diseases characterized by interactions between the gut, liver, and microbiota. Current discussions of the gut-liver axis often focus on the potential for gut-derived substances and microbiota-mediated signaling to influence liver pathology (25). Dysbiosis of gut microbiota can disrupt intestinal epithelial homeostasis and compromise the intestinal barrier, allowing bacterial components to enter the portal vein and systemic circulation. As a result, the liver is exposed to toxins, metabolites and by-products, which may trigger inflammation, cause hepatotoxicity, and directly contribute to liver carcinogenesis. Furthermore, gut microbiota is closely involved in the development of viral hepatitis and non-alcoholic steatohepatitis (NASH), both of which may lead to liver cirrhosis and significantly elevate the risk of HCC (26–28).

### 2.2 Mechanisms underlying gut microbiota-induced HCC initiation and progression

During the occurrence and development of HCC, different dietary structures, lifestyles and environmental factors can lead to changes in gut microbiota. Dietary cholesterol induced gut microbiota metabolites alteration including increased taurocholic acid and decreased 3-indolepropionic acid (29). A high-fiber diet can enrich beneficial microbiota and deplete potential detrimental ones in both humans and mice (30). Additionally, lifestyle changes also affect gut microbiota balance. Circadian rhythm disorders alter gut microbiota metabolic patterns, while increased stress responses elevate intestinal permeability (31). Furthermore, environmental factors such as air pollutants may interfere with the composition and function of gut microbiota through direct or indirect pathways, producing the carcinogenic metabolites (32). The following section will further elaborate on the mechanisms by which gut microbiota influences the initiation and development of HCC.

Gut microbiota plays a direct role in shaping the inflammatory microenvironment of the liver during HCC development and progression. By modifying the liver’s inflammatory status, gut microbiota can promote HCC progression. In Nlrp6-deficient mice, significant shifts in the microbiota were observed, characterized by a decrease in beneficial bacteria such as Akkermansia muciniphila and an increase in potentially harmful bacteria like Lactobacillus. These changes resulted in impaired intestinal barrier function, liver dysfunction and an elevated tumor burden. Harmful bacteria were found to facilitate HCC progression by activating neutrophil-mediated suppressive immune cells and impairing the function of CD8+ T cells, thereby disrupting the liver’s immune microenvironment (33, 34).

Gut dysbiosis is a key characteristic across all stages of chronic liver disease, and compromised intestinal barrier integrity is directly linked to the initiation and progression of HCC. When the intestinal barrier is disrupted, harmful substances—including bacteria, toxins, microbial metabolites and microbe-associated molecular patterns (MAMPs)—can translocate across the mucosal barrier, further aggravating dysbiosis (35, 36). This imbalance not only generates carcinogenic substances but also fosters liver fibrosis, cirrhosis and HCC progression by dysregulating immune responses, altering microbial metabolic pathways and increasing intestinal permeability (37–40).

Additionally, gut microbiota-derived metabolites have an significant impact on the initiation and progression of HCC (35). For example, a reduction in butyrate-producing bacteria can weaken the intestinal mucosal barrier, facilitating the progression of HCC (41). Besides, one study shows that butyrate supplementation specifically induces hepatocellular carcinoma cell apoptosis through activation of calcium signaling pathway and reactive oxygen species (ROS) generation. Significant inhibition of HCC proliferation and metastasis can be achieved through either butyrate supplementation or the depletion of SCAD gene (ACADS), which encodes a key enzyme for butyrate metabolism (42). Gut microbiota can also transform primary bile acids into secondary bile acids. Through the enterohepatic circulation, they then recirculated back to the liver (43). Secondary bile acids are not only hepatotoxic but also induce DNA damage and possess carcinogeni-c potential (26). In mouse models of oncogene-induced HCC and human HCC samples, studies utilizing gene expression profiling, metabolomic analysis and immunohistochemistry have identified SIRT5 as an important metabolic regulator in HCC development. It has been observed that SIRT5 expression is decreased in human HCC samples, while bile acid levels are elevated and correlated with increased M2 macrophage polarization. Another study shows that in mice, the loss of SIRT5, in conjunction with oncogenic signaling, leads to enhanced bile acid production due to hyper-succinylation in hepatocytes. Elevated bile acids serve as signaling molecules that activate the nuclear receptor FXR, promoting M2 macrophage polarization (44),In the TME, M2 macrophages are typically associated with tumor growth, invasion and metastasis. Research has shown that M2 macrophages release exosomes containing miR-23a-3p. These exosomes are then transferred to HCC cells and human umbilical vein endothelial cells. After being absorbed, the exosomes trigger several changes in these cells. They induce epithelial-mesenchymal transition (EMT), promote the formation of new blood vessels, and increase blood vessel permeability. Through these mechanisms, the exosomes ultimately enhance HCC metastasis. Moreover, the immunosuppressive properties of M2 macrophages enable tumor cells to evade immune surveillance, facilitating immune escape and further accelerating HCC progression (45). In liver cancer animal models, gut microbiota and TLR4 activation have been found to accelerate HCC progression by promoting cell proliferation and inhibiting apoptosis (46).

In conclusion, gut microbiota influences the liver’s inflammatory microenvironment through various direct and indirect mechanisms. The compromise of the intestinal barrier worsens the translocation of bacterial toxins and components, resulting in dysbiosis while increased levels of microbiota-derived metabolites, such as secondary bile acids, contribute to the initiation and progression of HCC.

### 2.3 Gut microbiota modulates antitumor immunity and affects cancer immunotherapy efficacy

Gut microbiota regulates liver immune responses through metabolites, including MAMPs, short-chain fatty acids and bile acids, which in turn modulate antitumor immunity (47, 48). One study has shown that gut microbiota-mediated bile acid metabolism can increase the abundance of CXCR6+ NKT cells in the liver, exerting antitumor effects in HCC. CXCL16 produced by liver sinusoidal endothelial cells attracts CXCR6+ NKT cells to this specific area, enhancing the presence of NKT cells in the liver and inhibiting tumor proliferation. A positive correlation has been observed between primary bile acid levels and CXCL16 expression, while secondary bile acids are negatively correlated (49).

In recent years, immunotherapy for HCC has made remarkable progress, with ICIs emerging as a primary therapeutic approach for advanced HCC. ICIs act by targeting co-inhibitory molecules such as PD-1/PD-L1, thereby enhancing T cell-mediated immune responses and preventing tumor immune evasion. Gut microbiota and its metabolites are essential in modulating T cell functions, profoundly affecting the efficacy of immunotherapy (50, 51). Microbial metabolites not only regulate antitumor immune responses but also potentiate the effectiveness of ICIs by reshaping the TME and modulating the immunogenicity of tumor cells.

Research has shown that oral administration of *Bifidobacterium* can enhance the maturation of dendritic cells (DCs) and increase the activation and accumulation of CD8+ T cells within the TME, thereby restoring the anticancer effectiveness of PD-L1 inhibitors. A study involving 65 patients with unresectable HCC or advanced cholangiocarcinoma, who switched to PD-1 inhibitors following first-line chemotherapy (gemcitabine plus cisplatin), indicated that the composition of gut microbiota is significantly related to the clinical responses of immunotherapy. In the clinical benefit group (CBR), 74 bacterial taxa were significantly enriched, whereas 40 taxa were enriched in the non-clinical benefit group (NCB). Patients in the CBR group with elevated levels of *Alistipes* sp. Marseille-P5997 and *Lachnospiraceae bacterium*-GAM79 exhibited longer overall survival (OS) and progression-free survival (PFS). Conversely, higher abundances of *Veillonellaceae* in the NCB group were associated with shorter PFS and OS (52). This study highlights the close link between gut microbiota and clinical responses to PD-1 inhibitors, suggesting that certain bacterial taxa could be potential biomarkers for predicting immunotherapy outcomes and survival benefits.

Gut microbiota synthesizes and converts small-molecule metabolites, such as inosine, short-chain fatty acids (SCFAs) and tryptophan, which circulate systemically to regulate antitumor immune responses. Inosine enhances tumor cell immunogenicity, activates immune cells and serves as a carbon source for CD8+ T cells, while SCFAs help maintain intestinal barrier integrity, inhibit tumor cell proliferation and induce apoptosis. Research has demonstrated that supplementation with *Lactobacillus* significantly suppresses HCC development in mice, primarily through the secretion of valeric acid and other SCFAs, thereby exerting antitumor effects in the liver (53). Furthermore, gut microbiota metabolite butyrate enhances the efficacy of cancer treatments by regulating intracellular calcium homeostasis and promoting the generation of ROS (42). Fecal microbiota composition and bile acid levels have also been correlated with the clinical response to immunotherapy in patients with unresectable HCC (54). In a study of 52 patients with solid tumors, higher concentrations of acetic acid, butyric acid, propionic acid and valeric acid in fecal samples showed longer progression-free survival (PFS), suggesting that SCFA levels may serve as potential biomarkers for predicting the efficacy of PD-1 inhibitors (55).

In summary, gut bacteria and their metabolites can affect the efficacy of immunotherapies such as ICIs, and some microbial communities may serve as predictive biomarkers for prognosis or treatment response in HCC patients (56). Additionally, gut microbiota may also influence the efficacy of other immunotherapies, such as adoptive cell transfer (ACT) therapy and cell-based therapies (57, 58).

## 3 Probiotics regulate gut microbiota to inhibit HCC growth

Research has demonstrated that dysbiosis promotes cancer progression by altering TME. Probiotics, due to their ability to modulate gut microbiota and enhance immune responses, have emerged as a new strategy to improve the effectiveness of cancer immunotherapy. They effectively inhibiting tumor growth, including HCC (59). Compared with traditional HCC treatments, probiotics exhibit unique characteristics in regulating anti-tumor immunity (**Table 1**). Probiotics significantly impact the gut-liver axis by regulating gut microbial composition, enhancing intestinal barrier integrity, modulating immune reactions and influencing key metabolic pathways (60–62, 73).

**Table 1:** Comparison of Mechanisms and Effects on Gut Microbiota between Probiotics and Conventional Treatments for HCC.

Treatment	Potential Immunomodulatory Mechanisms	Effects on Gut Microbiota	References
Probiotics	Increase CD8+ T cell infiltration Promote Th1 polarization Maintain intestinal barrier integrity Reduce inflammatory cytokines (IL-17, TNF-α)	Regulate gut microbial composition Enhance intestinal barrier integrity Restore microbial diversity	(6, 10, 60–65)
Surgery	Temporary immune suppression Increase inflammatory response	Dysbiosis	(66, 67)
Targeted Therapy	Enhance T cell-mediated tumor clearance Reduce immunosuppressive M2 macrophage infiltration	Metabolic influence	(68, 69)
ICIs	Enhance T cell activation Reverse the exhausted phenotype of tumor-infiltrating lymphocytes	Response correlation with microbiota	(9, 11, 65, 70)
Chemotherapy	Decrease CD8+ T cell number and function Increase TREM2+ TAM accumulation	Partially reverse the intestinal microflora disorder	(71, 72)

**Figure 2:**
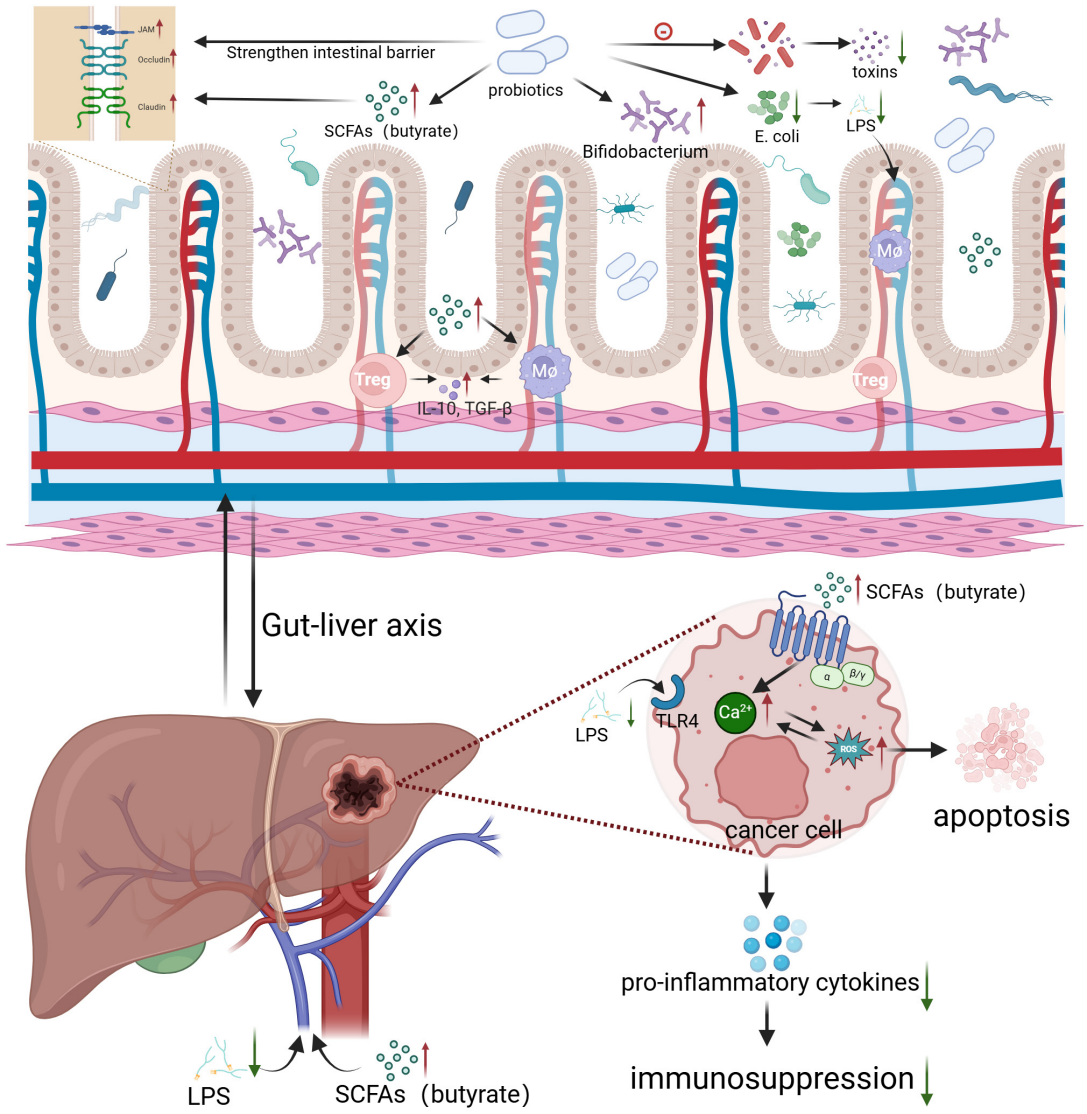
Probiotics regulate gut microbiota to inhibit HCC growth Created in BioRender. c, c. (2024) https://BioRender.com/f31p175.

Probiotics can reduce the risk of HCC through a variety of mechanisms (**Figure 2**). First, they regulate gut microbiota, preventing endotoxemia caused by dysbiosis and maintaining the integrity of the intestinal epithelial barrier, thus preventing the translocation of bacteria and MAMPs into the circulatory system. Additionally, probiotics produce helpful metabolites, which can alleviate oxidative stress in the liver of HCC patients by upregulating antioxidant enzyme expression (74, 75). Gut-derived lipopolysaccharide (LPS) can activate hepatic Kupffer cells, maintaining a pro-inflammatory and tumor-promoting environment via Toll-like receptor 4 (TLR4), which is often overexpressed in HCC tumor tissues. Probiotics protect the intestinal mucosa, reducing LPS translocation and thereby inhibiting HCC progression (76). A preclinical study demonstrated that fimbriae encoded by *Bifidobacterium bifidum* PRL2010 can induce the production of tumor necrosis factor-alpha (TNF-α). TNF-αplays an important part in both antitumor and anti-infective responses, it can promote immune cell communication without triggering inflammation (63). Prohep, a novel probiotic formulation, has been shown to significantly reduce tumor size and weight by approximately 40% in mouse models of HCC. In the TME, Th17 cells primarily secrete interleukin-17 (IL-17), a key factor in HCC progression and angiogenesis. Probiotics were found to significantly reduce the levels of IL-17 and Th17 cells, inhibiting tumor angiogenesis and resulting in tumor shrinkage. Immunohistochemistry revealed that the reduction in Th17 cells was primarily due to decreased migration from the gut and peripheral blood. This suggests that reducing Th17 cell recruitment and IL-17 secretion effectively impairs tumor angiogenesis and limits tumor growth. Probiotics also reshape gut microbiota. They promote the growth of beneficial bacteria such as *Spirochaetes* and *Prevotella*, which can produce anti-inflammatory metabolites, promote the differentiation of regulatory T cells (Treg/Tr1) and suppress Th17 polarization. In short, this research offers new insights into how probiotics modulate gut microbiota, regulate T cell differentiation, and influence pro-inflammatory factors in the TME, showing their preventive role in maintaining gut health (64). Additionally, a retrospective study of 1,267 chronic hepatitis B (HBC) patients found that patients who took ≥28 cumulative defined daily doses (cDDD) of probiotics had a lower risk of developing HCC compared to those who took <28 cDDD. The results suggested that probiotics might reduce the risk of HCC in CHB patients in a dose-dependent manner (77). Furthermore, another study has demonstrated that Bifidobacterium longum (BL) supplementation may improve postoperative outcomes in HCC patients through the enhancement of the tryptophan-5-hydroxytryptamine (5-HT) metabolic pathway, elevation of secondary bile acid levels and increase in SCFAs levels. These metabolic alterations were associated with improved liver function recovery and better clinical outcomes in HCC patients following surgery (78).

In conclusion, probiotics can facilitate a more favorable TME through modulation of the gut microbiota. By enhancing anti-tumor immune responses, probiotics represent a promising therapeutic strategy for HCC immunotherapy.

## 4 Related research advances

### 4.1 Current status of probiotics in HCC immunotherapy

Despite the remarkable efficacy of ICIs in HCC treatment, primary resistance to PD-1/PD-L1 blockade remains a significant challenge, with clinical benefit limited to only 15-20% of HCC patients (79–81). Recent studies have revealed that gut microbiota is closely associated with ICI efficacy, and its dysbiosis may be a key contributor to immunotherapy resistance (49, 60). At present, while clinical research on the use of probiotics in HCC immunotherapy remains relatively limited, modulating the gut microbiota through probiotics may represent a promising strategy to overcome ICI resistance and enhance immunotherapeutic outcomes.

### 4.2 Clinical evidence of gut microbiota modulation in cancer immunotherapy

Recent clinical studies have provided compelling evidence for the role of gut microbiota modulation in cancer immunotherapy. One phase I trial has shown that fecal microbiota transplantation(FMT)capsules derived from healthy donors, which represent another type of probiotics, are safe and can improve immune responses in melanoma patients who are refractory to PD-1 inhibitors. In responders, changes in gut microbiota composition following FMT were characterized by an enrichment of beneficial bacteria and a depletion of deleterious bacteria. This microbial modulation was associated with enhanced immune responses, including increased activated ICOS+CD8+ T cells and CD8+ MAIT cells, along with reduced immunosuppressive M-MDSCs. This novel therapeutic approach demonstrates safety and represents a promising treatment modality, though its clinical potential requires further evaluation in randomized clinical trials (65). Further evidence comes from a study analyzing clinical data from patients with non-small cell lung cancer (NSCLC), renal cell carcinoma (RCC) and urothelial carcinoma. This study compared the outcomes of patients who received antibiotics before and after ICI treatment with those who did not. The results demonstrated that regulating gut microbiota, particularly through the supplementation of beneficial bacteria like *Akkermansia muciniphila*, significantly improves the anticancer efficacy of PD-1/PD-L1 inhibitors. This suggests a promising strategy for incorporating probiotics as an adjunctive treatment in clinical practice. During immunotherapy, careful management of antibiotic use is essential to avoid compromising the efficacy of ICIs, when antibiotics are necessary, probiotics may aid in restoring gut microbiota, thereby maintaining therapeutic efficacy (9).These findings offer valuable insights for HCC immunotherapy, indicating that proper management of antibiotic use and regulation of gut microbiota could further enhance treatment outcomes.

Clinical observations have indicated that patients undergoing immunotherapy showed improvements in symptoms following adjunctive probiotic treatment, with significant enhancements in functional and quality-of-life scores. Besides, although ICIs have greatly advanced cancer therapy, their associated side effects remain a challenge, and modulation of gut microbiota could potentially help mitigate these adverse effects (82). However, there is a need for more extensive and rigorously designed studies to confirm these findings (83). There has been substantial exploration of probiotics use to treat other tumor types, and large amounts of related clinical trials are currently underway, particularly in the context of combining neoadjuvant chemotherapy with immunotherapy (**Table 2**). The clinical application of specific bacterial strains to regulate the microbiota in cancer treatment warrants further extensive investigation. If the “optimal” microbiota composition can be identified, it may be possible to modulate the patient’s gut microecology using specialized probiotics, prebiotics, or selective antibiotics. Overall, modulating gut microbiota shows great potential to enhance the antitumor effects of immunotherapy while mitigating treatment-related side effects. Probiotics hold promise as a safe and promising adjunctive strategy in the treatment of HCC, though continued research is essential to fully understand and optimize their therapeutic application (58, 76, 84).

**Table 2:** Clinical Trials Investigating the Effects of Probiotics in Combination with Anticancer Drugs for Cancer Therapy www.clinicaltrials.gov.

NCT number	Cancer types	n	Intervention	Stage
NCT03870607	Anal Cancer Squamous Cell	75	Prebiotics+ Probiotics + Radiotherapy+ Chemotherapy	Phase 2
NCT04699721	Non-small Cell Lung Cancer Stage III	40	Nivolumab +Paclitaxel(albumin-bound type) + Carboplatin AUC5 + BiFico	Phase 2
NCT03922035	Hematopoietic and Lymphoid Cell Neoplasm	36	Best Practice++Clostridium butyricum CBM 588 Probiotic Strain	Phase 1
NCT03829111	Renal Cell Carcinoma	30	Nivolumab+ Ipilimumab+ Clostridium butyricum CBM 588 Probiotic Strain	Phase 1
NCT05220124	Bladder Urothelial Carcinoma	190	Immunotherapy+ Live Combined (Bifidobacterium, Lactobacillus and Enterococcus Capsules)	Phase 4
NCT06039644	Breast Cancer	100	Chemotherapy+ Three-strain probiotics (Lactobacillus reuteri GMNL-89, Lactobacillus plantarum GMNL-141 and Lactobacillus paracasei GMNL-133)	Not Applicable
NCT06508034	Solid Malignancy	30	Immunotherapy+ Live Freeze-Dried Lactic Acid Bacteria Probiotic	Not Applicable
NCT06436976	Colorectal Cancer or Pancreatic Cancer	30	Lactobacillus reuteri ATG-F4	Phase 2
NCT05901779	Gastric Cancer	318	Neoadjuvant Chemotherapy+ Probiotics (Bifidobacterium longum, Lactobacillus acidophilus, and Enterococcus faecalis)	Not Applicable

## 5 Discussion

In the TME of HCC, the tumor immune barrier (TIB) obstructs the infiltration of immune cells like T cells, thereby diminishing the efficacy of immunotherapy. This finding offers valuable insights for the use of probiotics in HCC immunotherapy. By modulating the gut-liver axis and the immune system, probiotics may disrupt the TIB structure, thereby enhancing the cytotoxic activity of immune cells against tumors. Moreover, probiotics may reduce SPP1 secretion by influencing macrophage polarization, potentially alleviating TIB-mediated resistance to immunotherapy. Combining probiotics with ICIs (such as PD-1 inhibitors) could create synergistic effects, improving HCC patient responses to immunotherapy. Through personalized analysis of a patient’s gut microbiota and tumor immune microenvironment, probiotics have the potential to emerge as a critical adjunctive approach to boost immunotherapy efficacy (85).

Research has demonstrated a positive correlation between gut microbiota diversity and the efficacy of ICIs. Therefore, modulating gut microbiota—particularly by increasing diversity through probiotics—may help establish a more favorable microenvironment for HCC immunotherapy. This strategy not only enhances patient responses to ICIs but may also mitigate treatment-related side effects by promoting immune system balance. The potential of combining probiotics with ICIs, especially in patients unresponsive to current therapies, merits further investigation (56).

Moreover, gut microbiota signatures (Gut OncoMicrobiome Signatures, GOMS) are crucial for predicting both the efficacy and resistance of ICIs. By precisely modulating gut microbiota, it may be possible to optimize treatment regimens, thereby improving survival rates and clinical outcomes for HCC patients. In the future, the development and application of probiotics tailored to the individual gut microbiota profiles of patients could drive the implementation of personalized treatments, further enhancing the efficacy of HCC immunotherapy (86).

In recent years, the role of gut microbiota in liver-related diseases has been extensively explored. Through the gut-liver axis, gut microbiota impacts various stages of HCC development, including microbial dysbiosis, changes in microbial metabolites and immunomodulatory functions. The widespread use of probiotics in both non-tumorous and tumorous diseases has been well-documented, especially in the treatment of disorders affecting the digestive, neuropsychiatric, and circulatory systems. Currently, numerous clinical trials are underway, recruiting patients to investigate how probiotics can modulate gut microbiota to enhance antitumor immunity and improve the safety and efficacy of cancer immunotherapy.

Despite promising preclinical results in enhancing cancer immunotherapy efficacy, the application of probiotics in immunocompromised HCC patients raises several safety concerns that warrant careful consideration (51). These patients are more susceptible to infections, necessitating rigorous safety screening and monitoring of probiotic strains to prevent harmful genetic transfer to other bacteria. Additionally, probiotics intervention may lead to dysbiosis, causing gastrointestinal symptoms such as abdominal pain and diarrhea (87). Moreover, when combined with ICIs in HCC treatment, probiotics may also cause overactive immune responses, increasing the risk of immune-related adverse events (irAEs) and autoimmune reactions. To mitigate these risks in HCC immunotherapy, systematic preventive measures should be implemented, including pre-treatment immune function assessment, development of individualized probiotic dosing regimens, regular monitoring of gut microbiota changes and establishment of comprehensive adverse events early warning mechanisms. Future research should focus on optimizing the clinical application of probiotics in HCC immunotherapy through identification of novel strains, determination of optimal dosing strategies, rigorous safety assessments and large-scale clinical trials to document therapeutic efficacy (88). Except for the safety concerns, the translation of microbiota-based therapies from animal models to clinical applications in HCC immunotherapy faces several significant challenges (89). First, there are marked differences between animals and humans in gut microbiota composition, immune system complexity and gut-liver axis interactions. These differences may significantly impact therapeutic efficacy (90). Second, multiple technical challenges need to be addressed. These include standardization of probiotic production, quality control, optimization of delivery methods and maintenance of viability during storage and transportation. Third, clinical implementation must consider individual variations among patients, which include gut microbiota composition, immune responses, concurrent medications and dietary habits. Current clinical research has several limitations: There is a lack of standardized methods for microbiota analysis, clinical endpoint indicators are not unified across studies and Long-term safety data remains insufficient. Moreover, the precise mechanisms underlying probiotic-mediated immunomodulation require more comprehensive investigation (60, 91).

In spite of these challenges, the close connection between gut microbiota and the liver presents significant opportunities for microbiome-based approaches in HCC diagnosis, treatment and prevention (92). Based on previous findings in melanoma research, oral administration of microbiome capsules, a novel probiotic derived from fecal bacteria of either healthy donors or HCC patients who responded well to ICIs therapy, may enhance immunotherapy efficacy in HCC patients with suboptimal immune responses through modulation of the gut microbiota. Additionally, probiotics show promise in alleviating adverse effects associated with antineoplastic drugs, as well as promote liver function recovery. Future research should comprehensively monitor factors that modulate gut microbiota, including diet, medication, lifestyles, environment, the host immune system and population-specific microbiome variations across different geographical regions. To effectively evaluate the efficacy and safety of probiotics in HCC immunotherapy, it is essential to well design clinical trials that incorporate knowledge from microbiology, immunology, metabolomics, and epidemiology. Several key research priorities should be addressed. First, establishing standardized systems for probiotic screening and evaluation in collaboration with regulatory authorities is crucial for clinical translation. Second, developing innovative delivery systems and formulations could significantly improve bacterial colonization efficiency and therapeutic outcomes. Third, precision medicine approaches should be developed to select optimal bacterial combinations based on individual microbiome profiles, incorporating patient stratification strategies and artificial intelligence-driven bioinformatics analysis. Fourth, the molecular mechanisms by which probiotics modulate the tumor immune microenvironment need to be elucidated through multi-omics integration approaches. Finally, large-scale, multicenter randomized controlled trials with standardized protocols are needed to provide robust evidence supporting the clinical application of probiotics in HCC immunotherapy. In conclusion, probiotics demonstrate broad application prospects in HCC immunotherapy, yet require systematic research efforts and standardized approaches to fully realize their therapeutic potential in clinical practice.
